# Long-Term Mortality for Patients of Primary Aldosteronism Compared With Essential Hypertension: A Systematic Review and Meta-Analysis

**DOI:** 10.3389/fendo.2020.00121

**Published:** 2020-03-10

**Authors:** Zhe Meng, Zhe Dai, Kai Huang, Chang Xu, Yin-Gao Zhang, Hang Zheng, Tong-Zu Liu

**Affiliations:** ^1^Department of Urology, Zhongnan Hospital of Wuhan University, Wuhan University, Wuhan, China; ^2^Department of Endocrinology & Adrenal Hypertension Center, Zhongnan Hospital of Wuhan University, Wuhan University, Wuhan, China; ^3^Chinese Evidence Based Medicine Center, West China Hospital, Sichuan University, Chengdu, China

**Keywords:** primary aldosteronism, essential hypertension, mortality, systematic review, meta-analysis

## Abstract

**Background:** Consistent evidence have demonstrated that patients with primary aldosteronism (PA) have higher risk of cardiovascular events to patients with essential hypertension (EH). Whether the long-term risk of mortality for PA patients is higher than EH patients is unclear. We aim to compare the long-term mortality of patients with PA to patients with EH.

**Methods:** We searched PubMed, Embase, and Cochrane Central Register of Controlled Trials for eligible studies from inception to 14 Nov 2018. We combined the relative risks (RR) of each included study by random-effect model. The amount of between study heterogeneity was measured by the *I*^2^ statistic.

**Results:** We totally included six studies with cohort design, including 3,039 PA and 45,495 EH patients. The pooled RRs for patients with PA were 1.97 (95%CI: 1.33, 2.91; *P* = 0.0007) for a follow-up of 3 years, 0.96 (95%CI: 0.75, 1.23; *P* = 0.76) for 5 years, 0.86 (95%CI: 0.51, 1.46) for 7.5 years, and 0.95 (95%CI: 0.61, 1.48; *P* = 0.58) for 10 years. For patients with aldosterone-producing adenomas (APA), evidence of lower risk of long-term mortality was observed. Our sensitivity analysis suggested our results were stable.

**Conclusions:** Current evidence supported a higher risk of mortality for patients with primary aldosteronism at 3 years compared to patients with essential hypertension, however this risk no longer sustains as the follow-up time increased to 5 or more years. Patients with aldosterone-producing adenomas may have lower long-term mortality rate than patients with essential hypertension due to the better recovery of adrenalectomy.

## Introduction

Aldosterone is a type of steroid hormone produced by the adrenal cortex of the adrenal gland that plays an important role in maintaining the respective balances of blood pressure, fluids and electrolytes (e.g., Na^+^, K^+^) in the body (1). The secretion of aldosterone is primarily regulated by the renin-angiotensin-aldosterone system. Excessive production of aldosterone would lead to high levels of potassium in urinary excretion, which would then cause a series of internal milieu disorders, including renal artery contraction, hypokalemia, and metabolic alkalosis (2).

Primary aldosteronism (PA) is a condition that is a result of the autonomous excessive production aldosterone that escapes regulation from angiotensin or plasma potassium concentrations (3). PA is the main cause of secondary hypertension. It is estimated that, among patients with arterial hypertension, 5–10% of cases are caused by PA, with the percentage being much higher among patients with resistant hypertension (4, 5). Based on previous cross-sectional surveys, about half of the PAs were aldosterone-producing adenomas (APA) (6, 7).

Epidemiological studies (8–10) have shown a higher number of cases of cardiovascular disease, cerebrovascular disease, and metabolic disease among patients with PA than those with essential hypertension (EH). In a recent meta-analysis, during a median follow-up of 8.8 years, PA patients were shown to have a higher risk of stroke, coronary artery disease, heart failure, and diabetes than EH patients (11). This evidence suggested that there may be a poorer long-term prognosis for patients with PA than EH. However, regarding the long-term survival of PA and EH patients, the current findings are inconsistent and there has been no comprehensive evidence regarding the question of whether PA increase the risk of long-term mortality.

In this study, we conducted a systematic review and meta-analysis of cohort studies to investigate the long-term mortality for patients of PA and EH. We aim to provide comprehensive evidence of the effect of PA on long-term survival.

## Materials and Methods

This systematic review and meta-analysis have been reported according to the standard reporting guidance for meta-analysis of observational studies (12). This review has been submitted to PROSPERO for registration (CRD42019119125).

### Eligibility Criteria

Both prospective and retrospective cohort studies that had investigated the long-term mortality of PA patients compared with EH patients have been included in this study, regardless of the treatments involved with these patients. The population we focused on were adult PA patients who had both been treated and not been treated. The control group included adult EH patients. The primary outcome was “long-term mortality.” We defined long-term as at least 1 years. We included both full-text articles and informative abstracts published in English. We did not include studies without EH controls.

### Literature Search and Screen

We searched PubMed, Embase, and the Cochrane Central Register of Controlled Trials for eligible studies from inception to 14 Nov 2018. The search strategy was presented in Supplementary File. Two review authors (K. H. and C. X.) developed the search strategy based on MeSH terms and free text words in prior and then one review author (K. H.) conducted the literature search. Both of the two review authors were not librarians; instead, one author was a methodologist in the field of evidence-based medicine, and the other author was a Master's student of urology. A manual search was conducted by reviewing one published meta-analysis of PA and cardiovascular events (11). An updated search in PubMed was conducted in Jan-29 2020 and no additional studies were included.

The literature screen was conducted by two authors (Z. M. and K. H.). First, the titles and abstracts of the literature were reviewed by one author (K. H.) and those that did not explicitly meet the criteria were excluded. Then, the full texts of the remaining literature were reviewed by the two authors independently. Any disagreements were solved through the consensus of a core team of five experts (Z. M., K. H., T.-Z. L., Y.-G. Z., and H. Z.). For studies containing (but not reporting on) data that we were interested in, we contacted the main author to ask for potential data before deciding whether to include it or exclude it.

### Data Extraction

Data extraction was conducted by one author (K. H.) and then checked by another author (Z. M.). The following pieces of information, including article information, population, measurement, follow-up, and survival outcomes, were extracted: first authors' name, publication, region, demographic characteristics of population and controls (i.e., sample size, age, recruiting method), measurement of PA and EH, follow-up years, events and incidence rates of mortality during follow-up, and variables (controlled or balanced). For the studies that did not report detailed survival information, we extracted the data from the Kaplan-Meier curves using the Engauge digitizer software (version 10.9).

### Risk of Bias Assessment

Following the recommendation of the Cochrane handbook, we used the Newcastle-Ottawa Scale (NOS) to assess the risk of bias in the studies we included (13, 14). There were three domains referring to different type of biases: selection bias, confounding bias, and outcome measurement bias. Within each domain, there were two to four sub-items per bias, respectively, and there were nine sub-items for the scale in total. For each item, we assigned a score of 1 (Yes), 0.5 (Partial Yes), or 0 (No) to each study, depending on the extent to which the study met the requirements. We used the total score as a mark of the quality; a higher score indicating a lower risk of bias.

### Confounding Control

Given that hypertension is the main symptom of PA and EH, we referred to baseline systolic and diastolic blood pressure as the most important confounder of the outcome. In addition, age and comorbidity were shown to be two other important confounders in the current review, and has also been reported in previous studies. We used this definition as the criteria to assess the confounding bias in the risk of bias assessment. We extracted data controlled for the most variables to control the influence of confounding.

### Statistical Analysis

We used relative risk (RR) as an effect size estimator. The RR of each study was first calculated by extracting aggregate data. Then, a classical moment-based method based on a “two-stage” framework, with inverse variance as weight, was used to combine the RRs across the studies (13). The incidence rates of each group (i.e., PA group and EH group) were also combined separately. Taking the potential heterogeneity of the non-randomized trials we included into account, we employed a random-effect model to obtain a conservative estimate. The amount of heterogeneity was measured by the *I*^2^ statistic, which refers to the proportion of between-study variance against the total variance (15).

It should be noted that the outcome (death) of the current meta-analysis may be a rare event that, for small studies, may not occur in both groups. In such a situation, the classical “two-stage” meta-analytic method may exclude such studies directly and may lead to a potential estimation bias. Therefore, we employed a “one-stage” method based on multilevel logistic regression to re-combine the data as a sensitivity analysis to see if the results were stable (16).

A subgroup analysis was designed based on follow-up years (3, 5, 7.5, and 10 years), treatment methods of PA, and subtypes of PA (APA). The potential publication bias was visually judged by a funnel plot separated by each subgroup. When obvious asymmetry was observed, indicating evidence of publication bias, we used the nonparametric “trim and fill” method to adjust the effects (17). All the statistical analyses were conducted in the RevMan 5.3 software and R 3.5.1 software, with *P* < 0.05 as the statistical significance.

## Results

**Figure 1:**
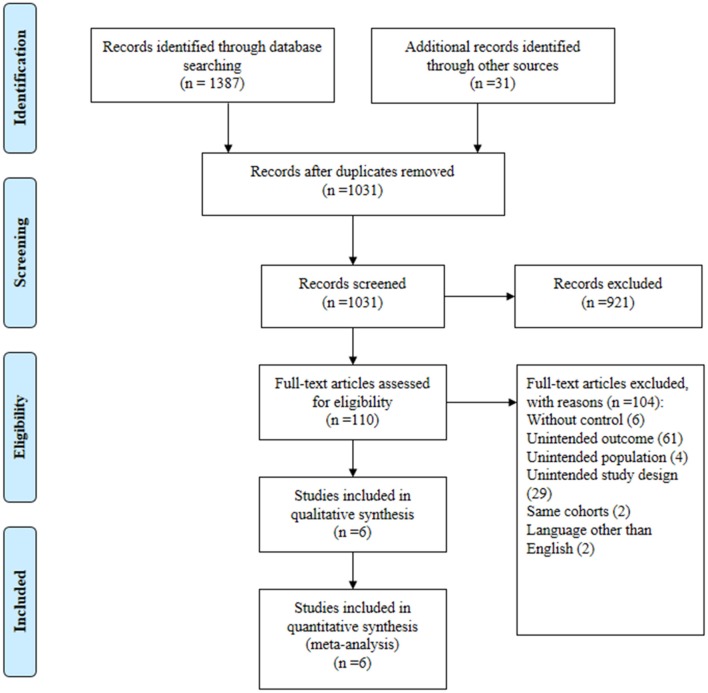
Flow plot of literature screen.

In total, 1,387 publications were obtained from the three databases, and 31 articles were collected from additional source. We identified 381 duplicates. From the remaining 1,031 publications, we excluded 921 based on their titles and abstracts. After reviewing the full texts of the 110 potential articles, we included six studies (18–23) in our systematic review and meta-analysis (Figure 1). We contacted the principal researcher of one study (9) for potential data, but there was no relevant data. The excluded references have been presented in a Supplementary File.

Of the six studies, three were published in 2018, and the earliest study was published in 2008. All of them were based on prospective or retrospective cohort design. Four of the studies were conducted in Europe, one in America, and one in Asia. The mean ages of the populations over the six studies were homogeneous, ranging from 46.2 to 53. All of the PA patients received surgical or medication treatment. The follow-ups ranged from 3 to 12 years across the studies. Quality scores ranged from 5.5 to 8.5, with a median score of 7. Important confounders were well controlled in only one study, partly controlled in two studies, but were not controlled in the other three studies. Table 1 presents the baseline characteristics and Table 2 presents the quality of the six studies.

**Table 1:** Baseline characteristics of include studies.

**References**	**Region**	**Population**	**Diagnose of PA and EH**	**Treatment of PA**	**Follow-up**	**Events and incidence**	**Adjusted or balanced variables**
Catena et al. (18)	Italy	54 consecutive patients (mean age: 53) who received a diagnosis of PA matched with 108 patients (mean age: 52) with EH	PA: increased plasma aldosterone–active renin ratio (≥20) EH: blood pressure 140/90 mmHg and/or current use of antihypertensive drugs	Unilateral adrenalectomy or spironolactone	7.4 years	PA: 0 (0%)	**Matching:** Age, sex, body mass index, duration of hypertension, systolic and diastolic blood pressure
						EH: 0 (0%)	
Reincke et al. (19)	German	281 PA patients (mean age: 50) matched with 281 EH patients (mean age: 50)	PA: increased plasma aldosterone–active renin ratio (≥20) EH: blood pressure 140/90 mmHg and/or current use of antihypertensive drugs	Unilateral adrenalectomy (47%) or mineralocorticoid receptor antagonists	10 years	PA: 32 (11.39%) EH: 37 (13.17%)	**Matching:** Age, sex, BMI, systolic and diastolic blood pressure
					5 years	PA: 11 (3.91%) EH: 15 (5.34%)	
					3 years	PA: 6 (2.14%)	
						EH: 6 (2.14%)	
Rossi et al. (23)	Italy	180 consecutive PA patients (mean age: 51.1) between 1992 and 2012, with 143 EH patients (mean age: 52.4) as controls; 148 of PA and 111 EH with followed data.	PA: standard guideline EH: No description	Unilateral adrenalectomy or mineralocorticoid receptor antagonists	3 years	PA: 1 (0.68%) EH: 1 (0.90%)	Crude
Chan et al. (20)	China	2,248 PA patients (mean age: 48.4) who ever used mineralocorticoid receptor antagonists, matched with 2,248 EH patients (mean age: 48.4)		Unilateral adrenalectomy or mineralocorticoid receptor antagonists	4.28 years	PA: 236 (10.50%) EH: 273 (12.14%)	**Matching:** Gender, age, baseline comorbidities, hypertensive drug, other concomitant medications
		875 APA patients (mean age: 46.62) matched with 875 EH patients (mean age: 46.31)	EH: blood pressure 140/90 mmHg and/or current use of antihypertensive drugs			APA: 28 (3.2%) EH: 84 (9.6%)	
Hundemer et al. (21)	United States	602 PA patients (mean age: 58) matched with 41,853 age matched EH patients (mean age: 57). Patients of PA who underwent surgical adrenalectomy, had a previous cardiovascular event, were not treated with MR antagonists		All treated with mineralocorticoid receptor antagonists	10 years	PA: 131 (65.17%)	**Matching:** Age, previous cardiovascular event
						EH: 21,700 (51.85%)	
					7.5 years	PA: 84 (22.39%)	
			EH: medical records (ICD-9: 401.0, 401.1, 401.9; ICD-10: I10, I11, I12, I13)			EH: 14,463 (18.55%)	
					5 years	PA: 45 (22.39%)	
						EH: 7,763 (18.55%)	
					2.5 years	PA: 19 (9.45%)	
						EH: 1,737 (4.15%)	
Rossi et al. (22)	Italy	41 APA patients (mean age: 50.9), 66 IPA patients (mean age: 49.6) and 894 EH patients (mean age: 46) for control	PA: increased plasma aldosterone–active renin ratio (≥40) at baseline and (≥30) postcaptopril administration	Unilateral adrenalectomy or mineralocorticoid receptor antagonists	12 years	PA: 66 (61.68%)	Balanced with gender, BMI, and Glomerular Filtration Rate (by baseline characteristics)
						EH: 595 (66.55%)	
					9 years	PA: 12 (11.21%)	
						EH: 152 (17.00%)	
					6 years	PA: 1 (0.93%)	
						EH: 15 (1.68%)	
					3 years	PA: 1 (0.93%)	
						EH: 7 (0.78%)	
			EH: blood pressure 140/90 mmHg and/or current use of antihypertensive drugs		12 years	APA: 25 (60.98%)	
						EH: 595 (66.55%)	
					9 years	APA: 5 (12.20%)	
						EH: 152 (17.00%)	
					6 years	APA: 0 (0%)	
						EH: 15 (1.68%)	
					3 years	APA: 0 (0%)	
						EH: 7 (0.78%)	

**Table 2:** Quality assessment.

**References**	**Domain 1: Selection**				**Domain 2: Comparability**		**Domain 3: Outcome**			**Total score**
	**Representativeness of the exposed cohort**	**Selection of the non-exposed cohort**	**Ascertainment of exposure**	**Outcome not present at start of study**	**Adjusted for most important confounders**	**Adjusted for second important confounders**	**Assessment of outcome**	**Follow-up long enough**	**Adequacy of follow up**	
Catena et al. (18)	Partial Yes	Yes	Yes	Yes	Yes	No	Yes	Yes	Yes	7.5
	**Reason:** Consecutive patients between January 1, 1994, and December 31, 2001	**Reason:** Essential hypertension recruited by frequency matching	**Reason:**1. prospective design; 2. with standard diagnose criteria	**Reason:** Outcome was death	**Reason:** Adjusted for baseline systolic and diastolic blood pressure	**Reason:** Did not control other important variables, such as comorbidity	**Reason:** Outcome was death	**Reason:** Follow-up was 7.4 years	**Reason:** 100% followed	
Reincke et al. (19)	Partial Yes	Yes	Yes	Yes	Yes	No	Yes	Yes	Yes	7.5
	**Reason:** Patients of the three largest German centers treated between 1994 and 2010	**Reason:** Essential hypertension recruited in the same centers	**Reason:**1. prospective design; 2. with standard diagnose criteria	**Reason:** Outcome was death	**Reason:** Adjusted for baseline systolic and diastolic blood pressure	**Reason:** Did not control other important variables, such as comorbidity	**Reason:** Outcome was death	**Reason:** Follow-up was 10 years or more	Reason: 100% followed	
Rossi et al. (23)	Partial Yes	Yes	Yes	Yes	No	No	Yes	Yes	NO	5.5
	**Reason:** Consecutive PA patients between 1992 and 2012	**Reason:** Essential hypertension recruited in the same centers	**Reason:** With standard diagnose criteria	**Reason:** Outcome was death	**Reason:** Did not adjusted for baseline systolic and diastolic blood pressure	**Reason:** Did not control other important variables	**Reason:** Outcome was death	**Reason:** Follow-up was 3 years	**Reason:** 82% PA and 78% EH patients followed	
Chan et al. (20)	Partial Yes	Yes	Yes	Yes	Yes	Yes	Yes	Yes	Yes	8.5
	**Reason:** The longitudinal database of Taiwan province of China	**Reason:** Essential hypertension recruited in the same source	**Reason:** With standard diagnose criteria and detailed medical records	**Reason:** Outcome was death	**Reason:** Adjusted for baseline systolic and diastolic blood pressure	**Reason:** Adjusted for comorbidity, sex, and concomitant medications	**Reason:** Outcome was death	**Reason:** Follow-up was 4.28 years	**Reason:** 100% followed	
Hundemer et al. (21)	Partial Yes	Yes	Yes	Yes	No	No	Yes	Yes	Yes	6.5
	**Reason:** Patients were recruited from two hospitals from 1991 and 2016 of USA	**Reason:** Essential hypertension recruited in the same hospitals	**Reason:** With standard diagnose criteria and detailed medical records	**Reason:** Outcome was death	**Reason:** Did not adjusted for baseline systolic and diastolic blood pressure	**Reason:** Did not control other important variables, such as comorbidity (except for CVD events)	**Reason:** Outcome was death	**Reason:** Follow-up more than 10 years	**Reason:** More than 98% were followed	
Rossi et al. (22)	Partial Yes	Yes	Yes	Yes	No	No	Yes	Yes	Partial Yes	6
	**Reason:** Consecutive patients between 2000 and 2005 in specialized centers of Italy	**Reason:** Essential hypertension recruited in the same centers	**Reason:** With standard diagnose criteria and detailed medical records	**Reason:** Outcome was death	**Reason:** Did not adjusted for baseline systolic and diastolic blood pressure	**Reason:** Did not control other important variables, such as comorbidity (except for CVD events)	**Reason:** Outcome was death	**Reason:** Follow-up more than 12 years	**Reason:** 89.0% followed	

### Long-Term Mortality of PA Compared With EH

**Figure 2:**
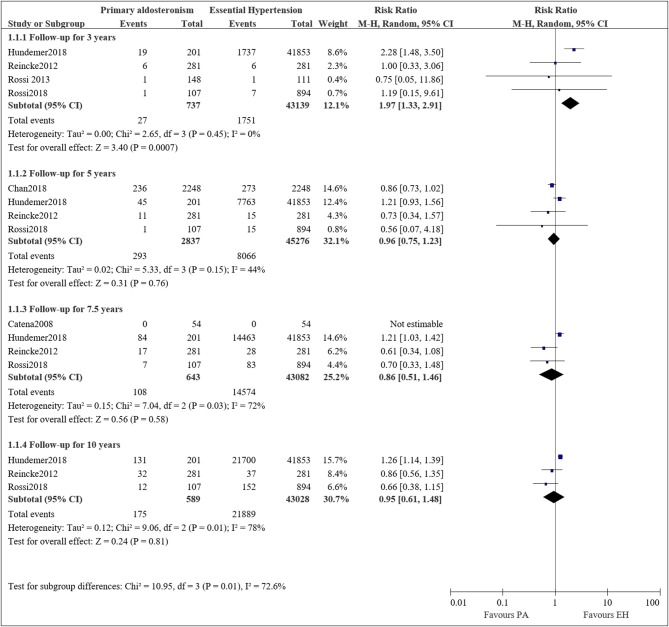
Comparison of patient with PA and EH of the risk of mortality at different follow-up times.

There were six studies that compared the long-term mortality of PA and EH patients. Figure 2 presents the pooled results of the risk of mortality at different follow-up times. Mortality after 3 years was available in four studies. In total, there were 737 PA patients with 27 death events, and 43,139 EH patients with 1,751 deaths. Our meta-analysis showed a higher risk of mortality for PA patients compared to EH (RR = 1.97, 95%CI: 1.33, 2.91; *P* = 0.0007); the between-study heterogeneity was mild (*I*^2^ = 0.0%).

The mortality rate at 5 years was available in four studies. In total there were 2,837 PA patients with 293 deaths and 45,276 EH patients with 8,066 deaths. The meta-analysis showed a comparable risk of mortality between the two groups (RR = 0.96, 95%CI: 0.75, 1.23; *P* = 0.76). A moderate heterogeneity was observed between the studies (*I*^2^ = 44%).

Mortality rates after 7.5 years were available in four studies, with one study reporting zero events in both groups. In total, there were 643 PA patients with 108 deaths and 43,190 EH patients with 14,574 deaths. The meta-analysis showed a lower risk of mortality in the PA group (RR = 0.86, 95%CI: 0.51, 1.46), while the results are statistically insignificant (*P* = 0.58). A substantial heterogeneity was observed between the studies (*I*^2^ = 72%).

Mortality at 10 years was available in three studies. In total, there were 589 PA patients with 175 deaths and 43,028 EH patients with 21,889 deaths. The meta-analysis showed a comparable risk of mortality between the two groups (RR = 0.95, 95%CI: 0.61, 1.48; *P* = 0.58). A substantial heterogeneity between the studies was observed (*I*^2^ = 78%).

### Long-Term Mortality of APA Compared With EH

**Figure 3:**
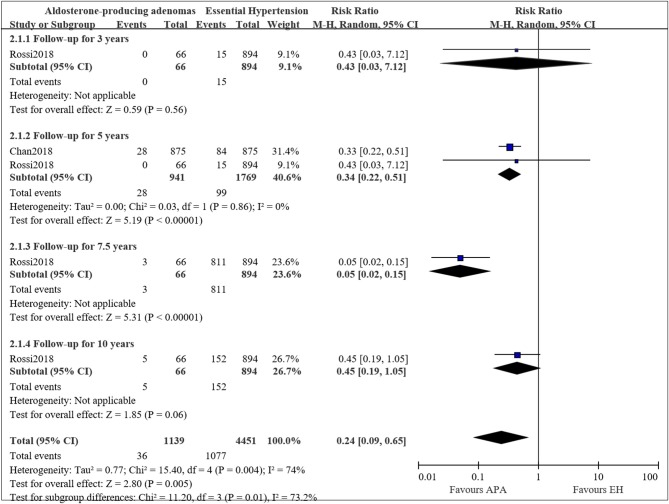
Comparison of patient with APA and EH of the risk of mortality at different follow-up times.

Two studies compared the long-term mortality between aldosterone-producing adenomas (APA) and EH patients, however, the data were only available for mortality at 5 years. Therefore, we pooled the results for mortality at 5 years. Figure 3 presents the pooled results, and some evidence of a lower mortality incidence in APA patients compared to EH was observed. The pooled results showed a decreased risk of mortality for APA patients compared to EH (RR = 0.34, 95%CI: 0.22, 0.51; *P* < 0.00001). Mild heterogeneity was observed (*I*^2^ = 0%).

The mortality at 3, 7.5, and 10 years were only available in the study by Rossi et al. (22), therefore we only described the result. In the study, RR for APA compared to EH was 0.43 (95%CI: 0.03, 7.12; *P* = 0.56) for a follow up of 3 years, 0.05 (95%CI: 0.02, 0.15; *P* < 0.00001) for a follow-up for 7.5 years and 0.45 (95%CI: 0.19, 1.05; *P* = 0.06) for a follow-up for 10 years.

### Long-Term Mortality of Medical Treated PA Compared With EH

**Figure 4:**
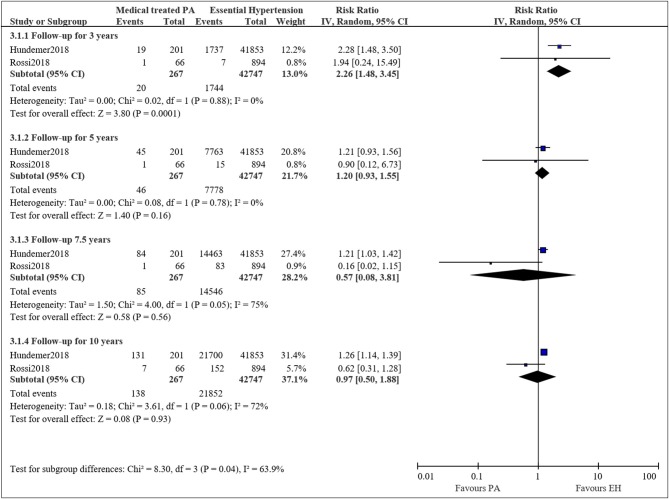
Comparison of patient with medical treated PA and EH of the risk of mortality at different follow-up times.

Two studies compared the long-term mortality for medical treated PA to EH patients (21, 22). The pooled RRs for PA patients treated with mineralocorticoid receptor antagonists compared to EH patients were 2.26 (95%CI: 1.48, 3.45; *P* < 0.001) at 3 years, 1.20 (95%CI: 0.93, 1.55; *P* = 0.16) at 5 years, 0.57 (95%CI: 0.08, 3.81; *P* = 0.56) at 7.5 years, and 0.97 (95%CI: 0.50, 1.88; *P* = 0.93) at 10 years (Figure 4). The results were consistent to the main analysis.

### The Long-Term Cumulative Mortality Rate and Cause of Death

**Figure 5:**
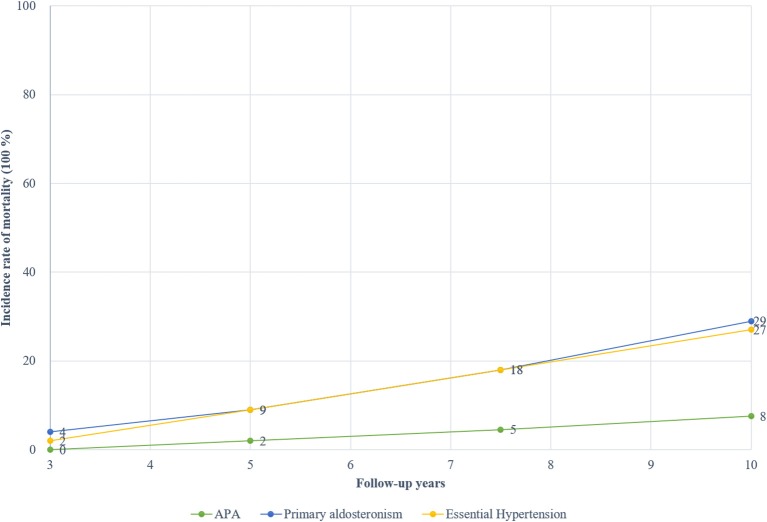
The absolute incidence of death of patient with PA and EH at different follow-up times.

Figure 5 presents the cumulative incidence rate for PA and EH according to the follow-up year. The pooled incidence rates of mortality for total PA stood at about 4% at 3 years, 9% at 5 years, 18% at 7.5 years, and 29% at 10 years. In contrast, the pooled incidence rates of mortality for matching EH patients were about 2% at 3 years, 9% at 5 years, 18% at 7.5 years, and 27% at 10 years. The mortality rates of APA were about 0% at 3 years, 2% at 5 years, 4.5% at 7.5 years, and 7.6% at 10 years. There was an obvious increasing trend for mortality rates based on the follow-up year. Only one study reported the mortality rates of idiopathic hyperaldosteronism, and were about 1.51% at 3, 5, and 7.5 years while 10.61% at 10 years (22).

For the cause of death, Reincke et al. reported 50% of which were due to cardiovascular diseases for PA patients (19); Rossi et al. reported a similar proportion that about 57% of the death for PA patients were caused by cardiovascular diseases (22). In the EH group, the cause of death by cardiovascular diseases was about 34% in Reincke's research (19) and 55% in Rossi's research (22).

### Sensitivity Analysis and Publication Bias

**Figure 6:**
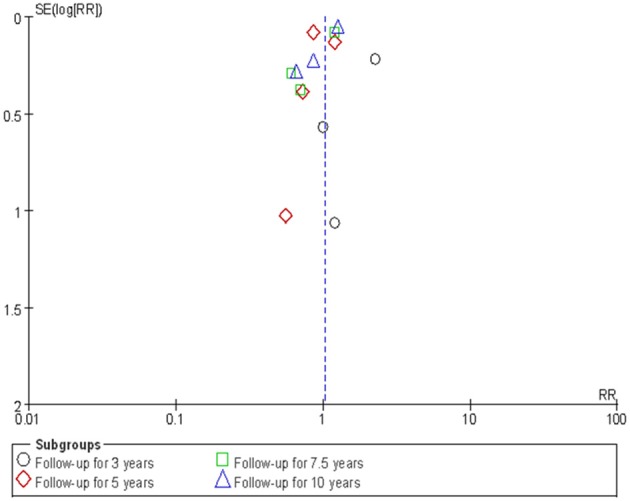
Publication bias.

The sensitivity analysis was conducted by employing a “one-stage” meta-analytic method to re-combine the results. Our re-analysis showed similar results between the “one-stage” method and the “two-stage” method we had previously used (Table 3). Figure 6 presents the funnel plot and there was some evidence of publication bias in the included studies. We then used the “trim and fill” method to adjust the influence of publication bias in the results. The results showed that the adjusted RRs resulting from the “trim and fill” method were similar to the “two-stage” method we used previously (Table 3).

**Table 3:** Sensitivity analysis.

**Sensitivity analysis**	**Long-term mortality of PA compared with EH**			
	**Follow-up 3 years**	**Follow-up 5 years**	**Follow-up 7.5 years**	**Follow-up 10 years**
Model A	1.97 (1.33, 2.91)	0.96 (0.75, 1.23)	0.86 (0.51, 1.46)	0.95 (0.61, 1.48)
Model B	1.91 (1.22, 3.00)	0.93 (0.74, 1.18)	0.91 (0.55, 1.49)	1.03 (0.62, 1.72)
Model C	1.97 (1.33, 2.91)	0.96 (0.75, 1.23)	0.87 (0.52, 1.44)	0.96 (0.64, 1.43)

*Model A: Classical “two-stage” meta-analysis*.

*Model B: “One-stage” meta-analysis that included the study reported zero events in both groups*.

*Model C: “Trim and Fill” method that adjusted the potential publication bias*.

## Discussion

We conducted a systematic review and meta-analysis to compare the long-term risk of mortality for PA patients and EH patients. Our findings suggested that, compared to EH patients, there was a higher risk of death for PA patients after a follow-up of 3 years, while after a follow-up for 5 years or more, the risks of death were comparable between the two groups. The incidence rates of both the PA and EH patients increased as the follow-up year increased, with the 10-year cumulative incidence rate at about 27–29%. In the subgroup analysis, we found that for APA patients, the risk of long-term mortality was lower than EH patients.

We observed that in the study by Hundemer et al. (21), PA patients who had received surgical treatment were all excluded so that the study only included PA patients treated with mineralocorticoid receptor antagonists. In the study by Rossi et al. (22), a subgroup with patients of idiopathic hyperaldosteronism that treated medically was compared to EH. We noticed that in Rossi's et al. study (22) the mortality incidence of medical treated PA was higher at 3 years while lower when follow-up time increased to 5 or more years compared to EH. This is likely due to insufficient sample size that only 66 PA patients received medical treatment and the event (*n* = 1) kept unchanged until followed for 9 years. This is why the RR was 0.16 at 3 years but increased to 0.63 at 10 years. The pooled results suggested that medical treated PH showed higher risk of mortality after a follow-up for 3 years (while this risk no longer sustains as the follow-up time increased to 5 or more years). Actually, this pooled result is mainly dominated by Hundemer's et al. study (21). And this indicated that more studies are warranted to verify if this result is of stable. The other four studies included PA patients who were both treated by surgery and/or mineralocorticoid receptor antagonists. Longitudinal studies and meta-analyses have compared the long-term outcomes of surgical and medical treatments for patients with PA (24–26). Their findings suggested that for patients with PA, surgical treatment leads to better hypertension and hypokalemia outcomes compared to medical treatment. In the results of our subgroup analysis, APA patients have a lower risk of death than EH patients. In fact, APA patients generally received the adrenalectomy treatment and this may be the reason why APA patients showed favorable survival.

The long-term clinical outcomes have been investigated in numerous epidemiological studies. Consistent evidence has demonstrated that there is a significant relationship between PA and adverse clinical outcomes, especially for cardiovascular events, cerebrovascular events, and target organ damage (e.g., renal impairment) compared to EH patients (8–11). The meta-analysis by Monticone et al. (12) confirmed that there was a higher cardiovascular risk for patients with PA than EH. These major adverse clinical outcomes may further lead to a higher risk of death. In our meta-analysis, however, we only observed a potential higher risk of mortality for overall PA but not for subtypes of PA, and patients with APA may face a better outcome than patients with EH. One possible explanation for this is that for patients with APA, an adrenalectomy is the optimal treatment which has shown better hypertension and hypokalemia outcomes compared to medical treatments (5, 26).

The goal of the treatment of PA is to reverse the hypokalemia and normalize the hypertensive status to keep it at a normalized level (27). Surgical and medical treatments are two effective treatments for PA patients, the selection of which depends on the cause of the PA (e.g., location, gene types) (28). For patients with unilateral PA or APA, surgical management is the optimal treatment, while for patients with bilateral PA, mineralocorticoid receptor antagonists were recommended as the optimal treatment (5, 27, 28). In our meta-analysis, we found that APA patients have a lower risk of death compared with EH patients, but not compared with overall PA patients. This may suggest that treatment approaches may influence long-term outcomes. In the current systematic review, however, we did not have further data in order to compare the long-term mortality of PA patients with different treatment approaches to EH patients. Further studies are required to explore this hypothesis.

There were several strengths to the current systematic review and meta-analysis. To the best of our knowledge, this is currently the first meta-analysis that has compared the long-term mortality incidence of patients with PA to patients with EH. Our systematic review shall provide comprehensive evidence to guide clinical practice. In addition, the current systematic review and meta-analysis was based on strict design and conduct according to standard guidance, which ensures a high quality. Moreover, we employed a comprehensive statistical procedure to synthesize the findings of the included studies, resulting in our results being more unbiased. However, there were also some limitations. First, there were limited available studies included in the current study, though we did carry out a comprehensive literature search. Second, all the included studies were based on observational design, and only one was adjusted toward the most important confounders we that we had previously defined. The pooled results may have been influenced by potential bias. Third, the follow-up time differs across studies and some of them failed to follow up for more than 5 years, which would lead to selection bias and make the conclusion unstable. Based on these limitations, further studies are still required to verify the findings.

## Conclusion

In conclusion, evidence from the current systematic review and meta-analysis supported a higher risk of mortality for patients with primary aldosteronism at 3 years compared to patients with essential hypertension, however this risk no longer sustains as the follow-up time increased to 5 or more years. Owing to the better recovery of surgical treatment, patients with aldosterone-producing adenomas may have a lower long-term mortality rate than patients with essential hypertension.

## Data Availability Statement

The raw data supporting the conclusions of this article will be made available by the authors, without undue reservation, to any qualified researcher.

## Author Contributions

ZM, ZD, and T-ZL proposed the ideal. KH searched and screened the literature, collect data, and analyzed the data. ZM and T-ZL assessed the quality and drafted the manuscript. CX contributed the search strategy development and the statistical analysis methods. ZD, Y-GZ, and HZ participated the literature screen and revised the manuscript.

### Conflict of Interest

The authors declare that the research was conducted in the absence of any commercial or financial relationships that could be construed as a potential conflict of interest.
