# Supplementary material for: Long-Term Mortality for Patients of Primary Aldosteronism Compared With Essential Hypertension: A Systematic Review and Meta-Analysis
Source: Front Endocrinol (Lausanne). 2020 Mar 10;11:121. doi: 10.3389/fendo.2020.00121 (PMC7075813; doi:10.3389/fendo.2020.00121)
Supplement: Supplementary file 1 [file Data_Sheet_1.DOCX]

**Search Strategy**

**EMBASE and Cochrane Central Register of Controlled Trials**

1. Hyperaldosteronism/
2. Primary Hyperaldosteronism.ti,ab
3. Primary aldosteronism.ti,ab
4. Aldosteronism.ti,ab
5. Conn* adj1 Syndrome.ti,ab
6. 1 OR 2 OR 3 OR 4 OR 5
7. Death/
8. Mortaliy.mp
9. Outcome*.mp
10. 7 OR 8 OR 9
11. 6 AND 10

**PubMed**

(((("hyperaldosteronism"[MeSH Terms] OR Primary Hyperaldosteronism[Title/Abstract]) OR Primary aldosteronism[Title/Abstract]) OR Aldosteronism[Title/Abstract]) OR Conn Syndrome[Title/Abstract]) AND (("death"[MeSH Terms] OR Mortaliy[Title/Abstract]) OR Outcome*[Title/Abstract])

**Updated search (PubMed)**

(("2018/11/01"[Date - Publication] : "3000"[Date - Publication])) AND ((((("hyperaldosteronism"[MeSH Terms] OR Primary Hyperaldosteronism[Title/Abstract]) OR Primary aldosteronism[Title/Abstract]) OR Aldosteronism[Title/Abstract]) OR Conn Syndrome[Title/Abstract]) AND (("death"[MeSH Terms] OR Mortaliy[Title/Abstract]) OR Outcome*[Title/Abstract]))

**Studies excluded**

1. Anonymous (2006). "Erratum: Long-term renal outcomes in patients with primary aldosteronism (Journal of American Medical Association (2006) 295 (2638-2645))." Journal of the American Medical Association 296(15): 1842.
2. Au, C. F., et al. (2018). "Adrenalectomy attenuates the risk of end stage renal disease of primary aldosteronism-a competing risk analysis." Journal of Urology 1994(Supplement 1): e24.
3. Baey, S., et al. (2018). "Timing of surgical intervention has an impact on cure of endocrine hypertension: Results from a tertiary referral center in South East Asia." World Journal of Endocrine Surgery 10(1): 45-46.
4. Blumenfeld, J. D., et al. (1994). "Diagnosis and treatment of primary hyperaldosteronism." Annals of Internal Medicine 121(11): 877-885.
5. Boulkroun, S., et al. (2012). "Prevalence, clinical, and molecular correlates of KCNJ5 mutations in primary aldosteronism." Hypertension 59(3): 592-598.
6. Catena, C., et al. (2007). "Relationships of plasma renin levels with renal function in patients with primary aldosteronism." Clinical journal of the American Society of Nephrology : CJASN 2(4): 722-731.
7. Catena, C., et al. (2006). "Insulin sensitivity in patients with primary aldosteronism: A follow-up study." Journal of Clinical Endocrinology and Metabolism 91(9): 3457-3463.
8. Celen, O., et al. (1996). "Factors influencing outcome of surgery for primary aldosteronism." Archives of Surgery 131(6): 646-650.
9. Chiou, T. T. Y., et al. (2009). "Factors determining cardiovascular and renal outcomes after adrenalectomy in patients with aldosterone-producing adrenal adenoma." Tohoku Journal of Experimental Medicine 218(1): 17-24.
10. Chiu, C. C., et al. (2013). "Predictors of the long-term outcomes in patients with secondary hypertension caused by primary aldosteronism." Circulation 128(22 SUPPL. 1).
11. Dobrucki, T., et al. (2006). "Treatment effects in primary hyperaldosteronism." Nadcisnienie Tetnicze 10(5): 392-402.
12. Fourkiotis, V., et al. (2013). "Effectiveness of eplerenone or spironolactone treatment in preserving renal function in primary aldosteronism." European Journal of Endocrinology 168(1): 75-81.
13. Fu B, et al. (2011). "Long-term results of a prospective, randomized trial comparing retroperitoneoscopic partial versus total adrenalectomy for aldosterone producing adenoma." Journal of Urology 185(5): 1578.
14. Furuta, N., et al. (2013). "The prevalence of hypertension after adrenalectomy in patients with primary aldosteronism." Acta Urologica Japonica 59(4): 225-229.
15. Giacchetti, G., et al. (2008). "Primary aldosteronism, a major form of low renin hypertension: from screening to diagnosis." Trends in Endocrinology and Metabolism 19(3): 104-108.
16. Giacchetti, G., et al. (2007). "Aldosterone as a key mediator of the cardiometabolic syndrome in primary aldosteronism: An observational study." Journal of Hypertension 25(1): 177-186.
17. Gockel, I., et al. (2007). "Long-term results of endoscopic adrenalectomy for Conn's syndrome." American Surgeon 73(2): 174-180.
18. Grytaas, M. A., et al. (2016). "Clinical outcome after treatment of primary aldosteronism in western norway." Endocrine Reviews 37(2 Supplement 1).
19. Grytaas, M. A., et al. (2017). "Clinical Characteristics and Long-Term Outcome of Primary Aldosteronism in a Norwegian Population." Hormone and Metabolic Research 49(11): 838-846.
20. Henry, J. F., et al. (2000). "Complications of laparoscopic adrenalectomy: Results of 169 consecutive procedures." World Journal of Surgery 24(11): 1342-1346.
21. Herd, A., et al. (2010). "Surgical outcomes following laparoscopic adrenalectomy for treatment of Conn's syndrome (primary hyperaldosteronism) between 1999 and 2006." New Zealand Medical Journal 123(1324): 50-56.
22. Hundemer, G., et al. (2018). "Treatment of primary aldosteronism and incidence of atrial fibrillation." Endocrine Reviews 39(2 Supplement 1).
23. Hundemer, G. L., et al. (2018). "Incidence of atrial fibrillation and mineralocorticoid receptor activity in patients with medically and surgically treated primary aldosteronism." JAMA Cardiology 3(8): 768-774.
24. Ishidoya, S., et al. (2011). "Single center outcome of laparoscopic adrenalectomy for patients with unilateral aldosterone excess: Lateralizing disease based on the results of adrenal venous sampling." Journal of Urology 185(4 SUPPL. 1): e10.
25. Iwakura, Y., et al. (2011). "Clinical significance of early diagnosis of primary aldosteronism to prevent causing and progressing renal dysfunction - The existence of quite a few patients with 'masked chronic kidney disease' disguising normal renal function before the specific therapy - The e." Hypertension 58(5): e120-e121.
26. Jeck, T., et al. (1994). "Primary aldosteronism: Difference in clinical presentation and long-term follow-up between adenoma and bilateral hyperplasia of the adrenal glands." Clinical Investigator 72(12): 979-984.
27. Jonsdottir, G., et al. (2017). "Primary aldosteronism: from case detection to histopathology with up to 6 years of follow-up." Journal of Clinical Hypertension 19(4): 424-430.
28. Katabami T, et al. (2017). "Cardiovascular and renal outcomes in unilateral primary hyperaldosteronism: comparative study between adrenalectomy and medical treatment." Endocrine reviews. Conference: 99th annual meeting of the endocrine society, ENDO 38(3 Supplement 1) (no pagination).
29. Kawasaki, Y., et al. (2016). "Medium-term outcomes after laparoscopic adrenalectomy for primary aldosteronism." Journal of Urology 195(4 SUPPL. 1): e127.
30. Kim, D. H., et al. (2016). "Risk factors for renal impairment revealed after unilateral adrenalectomy in patients with primary aldosteronism." Medicine (United States) 95(27): e3930.
31. Lo, C. Y., et al. (1996). "Primary aldosteronism: Results of surgical treatment." Annals of Surgery 224(2): 125-130.
32. Loh, H. H., et al. (2018). "Improvement of bone turnover markers and bone mineral density following treatment of primary aldosteronism." Minerva endocrinologica 43(2): 117-125.
33. Maiolino, G. P., et al. (2016). "Long-term outcome of surgically-and medically-treated primary aldosteronism (PA) in the PA Prevalence in hYpertensives (PAPY) Study." European Heart Journal 37(Supplement 1): 1228.
34. Markou A, et al. (2013). "Evidence of primary aldosteronism in a predominantly female cohort of normotensive individuals: a very high odds ratio for progression into arterial hypertension." The Journal of clinical endocrinology and metabolism 98(4): 1409.
35. Massien-Simon, C., et al. (1995). "Primary aldosteronism. Diagnostic and prognostic value of potassium, renin, aldosterone and the aldosterone/renin ratio." Presse Medicale 24(27): 1238-1242.
36. Middeke, M. and J. Schrader (1994). "Nocturnal blood pressure in normotensive subjects and those with white coat, primary, and secondary hypertension." British Medical Journal 308(6929): 630-632.
37. Miller, B. S., et al. (2018). "Refining the Definitions of Biochemical and Clinical Cure for Primary Aldosteronism Using the Primary Aldosteronism Surgical Outcome (PASO) Classification System." World Journal of Surgery 42(2): 453-463.
38. Miyake, Y., et al. (2014). "Prognosis of primary aldosteronism in japan: Results from a nationwide epidemiological study." Endocrine Journal 61(1): 35-40.
39. Muiesan, M. L., et al. (2008). "Inappropriate left ventricular mass in patients with primary aldosteronism." Hypertension 52(3): 529-534.
40. Mulliez, E., et al. (1998). "Long term results of unilateral adrenalectomy in primary aldosteronism. 91 Patients." Revue Francaise d'Endocrinologie Clinique - Nutrition et Metabolisme 39(3): 257-267.
41. Nakada, T., et al. (1995). "Therapeutic outcome of primary aldosteronism: Adrenalectomy versus enucleation of aldosterone-producing adenoma." Journal of Urology 153(6): 1775-1786.
42. Namekawa, T., et al. (2017). "Hypertension Cure Following Laparoscopic Adrenalectomy for Hyperaldosteronism is not Universal: Trends Over Two Decades." World Journal of Surgery 41(4): 986-990.
43. Nanba, A. T., et al. (2017). "Discordance between imaging and immunohistochemistry in primary aldosteronism." Endocrine Reviews 38(3 Supplement 1).
44. Nogueira-Silva, L., et al. (2015). "Deciphering the role of vasopressin in primary aldosteronism." Journal of Clinical Endocrinology and Metabolism 100(9): 3297-3303.
45. Novello, M., et al. (2007). "Renal cysts and hypokalemia in primary aldosteronism: Results of long-term follow-up after treatment." Journal of Hypertension 25(7): 1443-1450.
46. Obara, T., et al. (1992). "Risk factors associated with postoperative persistent hypertension in patients with primary aldosteronism." Surgery 112(6): 987-993.
47. Ohno, Y., et al. (2018). "Obesity as a Key Factor Underlying Idiopathic Hyperaldosteronism." The Journal of clinical endocrinology and metabolism 103(12): 4456-4464.
48. Okato, A., et al. (2013). "Examination about the laparoscopic partial adrenalectomy for primary aldosteronism." Journal of Urology 189(4 SUPPL. 1): e20-e21.
49. Park, K. S., et al. (2017). "Outcomes analysis of surgical and medical treatments for patients with primary aldosteronism." Endocrine Journal 64(6): 623-632.
50. Parthasarathy, H. K., et al. (2011). "A double-blind, randomized study comparing the antihypertensive effect of eplerenone and spironolactone in patients with hypertension and evidence of primary aldosteronism." Journal of Hypertension 29(5): 980-990.
51. Pokrovskii, A. V., et al. (2009). "The long-term follow up review of patients with primary hyperaldosteronism after unilateral portalisation of adrenal and renal blood." Khirurgiia(3): 65-66.
52. Proye, C. A. G., et al. (1998). "Essential hypertension: First reason for persistent hypertension after unilateral adrenalectomy for primary aldosteronism?" Surgery 124(6): 1128-1133.
53. Puccini, M., et al. (1998). "Conn syndrome: 14 year's experience from two European centres." European Journal of Surgery 164(11): 811-817.
54. Quarmby, C. J., et al. (1995). "Conn's syndrome due to adrenocortical adenoma - A rare but rewarding cause of curable hypertension." South African Medical Journal 85(12 II): 1353-1356.
55. Reincke, M., et al. (2009). "Risk factors associated with a low glomerular filtration rate in primary aldosteronism." Journal of Clinical Endocrinology and Metabolism 94(3): 869-875.
56. Rossi, G. P., et al. (2006). "A Prospective Study of the Prevalence of Primary Aldosteronism in 1,125 Hypertensive Patients." Journal of the American College of Cardiology 48(11): 2293-2300.
57. Roukounakis, N., et al. (2007). "Is preservation of the adrenal vein mandatory in laparoscopic adrenal-sparing surgery?" JSLS : Journal of the Society of Laparoendoscopic Surgeons / Society of Laparoendoscopic Surgeons 11(2): 215-218.
58. Rutherford, J. C., et al. (1998). "Success of surgery for primary aldosteronism judged by residual autonomous aldosterone production." World Journal of Surgery 22(12): 1243-1245.
59. Saint, F., et al. (2017). "Long-term blood pressure outcome after unilateral adrenalectomy for primary hyperaldosteronism." Progres en urologie : journal de l'Association francaise d'urologie et de la Societe francaise d'urologie 27(6): 389-394.
60. Sechi, L. A., et al. (2006). "Long-term renal outcomes in patients with primary aldosteronism." Journal of the American Medical Association 295(22): 2638-2645.
61. Sharma, B. K., et al. (1994). "Malignant hypertension in north west India. A hospital based study." Japanese Heart Journal 35(5): 601-609.
62. Shevchenko, I. L., et al. (2003). "Current aspects of diagnosis and treatment of symptomatic arterial hypertension of adrenal genesis." Terapevticheskii arkhiv 75(4): 8-15.
63. Sim, J. J., et al. (2011). "Aldosterone to renin ratio (ARR) as a predictor of mortality and cardiovascular outcomes." Journal of Clinical Hypertension 13(4 SUPPL. 1): A66-A67.
64. Siren, J., et al. (1998). "Adrenalectomy for primary aldosteronism: Long-term follow-up study in 29 patients." World Journal of Surgery 22(4): 418-422.
65. Strauch, B., et al. (2017). "Long-term effects of adrenalectomy or spironolactone on arterial stiffness in primary aldosteronism." Journal of Hypertension 35(Supplement 2): e58.
66. Sukor, N., et al. (2009). "Role of unilateral adrenalectomy in bilateral primary aldosteronism: A 22-year single center experience." Journal of Clinical Endocrinology and Metabolism 94(7): 2437-2445.
67. Sukor, N., et al. (2010). "Improved quality of life following unilateral laparoscopic adrenalectomy in patients with unilateral primary aldosteronism." Hypertension 55(6): 1509-1510.
68. Sy, W. M., et al. (2012). "Primary hyperaldosteronism among Chinese hypertensive patients: How are we doing in a local district in Hong Kong." Hong Kong Medical Journal 18(3): 193-200.
69. Van Der Linden, P., et al. (2010). "Blood pressure reduction following adrenalectomy in primary aldosteronism: A retrospective follow-up study." Journal of Hypertension 28(SUPPL. A): e301-e302.
70. van der Linden, P., et al. (2012). "Blood pressure and medication changes following adrenalectomy for unilateral primary aldosteronism: A follow-up study." Journal of Hypertension.
71. Vasan, R. S., et al. (2004). "Serum aldosterone and the incidence of hypertension in nonhypertensive persons." New England Journal of Medicine 351(1): 33.
72. Velema M, D. T. H. A. T. H. L. J. G. H. S. K. L. L. J. P. A. v. d. W. G. J. D. J. (2018). "Quality of Life in Primary Aldosteronism: a Comparative Effectiveness Study of Adrenalectomy and Medical Treatment." Journal of Clinical Endocrinology and Metabolism 103(1): 16.
73. Volpe, C., et al. (2015). "Primary aldosteronism: Functional histopathology and long-term follow-up after unilateral adrenalectomy." Clinical Endocrinology 82(5): 639-647.
74. Waldmann, J., et al. (2011). "Outcome of surgery for primary hyperaldosteronism." World Journal of Surgery 35(11): 2422-2427.
75. Weigel, R. J., et al. (1994). "Surgical treatment of primary hyperaldosteronism." Annals of Surgery 219(4): 347-352.
76. Williams, T. A., et al. (2017). "Outcomes after adrenalectomy for unilateral primary aldosteronism: an international consensus on outcome measures and analysis of remission rates in an international cohort." The Lancet Diabetes and Endocrinology 5(9): 689-699.
77. Wu, V. C., et al. (2016). "Long term outcome of Aldosteronism after target treatments." Scientific reports 6: 32103.
78. Zarnegar, R., et al. (2007). "Good blood pressure control on antihypertensives, not only response to spironolactone, predicts improved outcome after adrenalectomy for aldosteronoma." Surgery 142(6): 921-929.
79. Zhang, X., et al. (2013). "Factors affecting complete hypertension cure after adrenalectomy for aldosterone-producing adenoma: outcomes in a large series." Urol Int 90(4): 430-434.
80. Fischer. E., et al. (2013). “Aldosterone excess impairs first phase insulin secretion in primary aldosteronism.” J Clin Endocrinol Metab. 98(6):2513-20.
81. Curione. M., et al. (2014). “Electrical and Myocardial Remodeling in Primary Aldosteronism.” Front Cardiovasc Med. 6;1:7.
82. Fallo F. Prevalence and characteristics of the metabolic syndrome in primary aldosteronism. J Clin Endocrinol Metab. 2006 Feb;91(2):454-9.
83. Goldkorn R. Echocardiographic comparison of left ventricular structure and function in hypertensive patients with primary aldosteronism and essential hypertension. Am J Hypertens. 2002 Apr;15(4 Pt 1):340-5.
84. Hung CS. Twenty-four-hour urinary aldosterone predicts inappropriate left ventricular mass index in patients with primary aldosteronism. ScientificWorldJournal. 2013 Dec 29;2013:294594.
85. Iacobellis G. Adipokines and cardiometabolic profile in primary hyperaldosteronism. J Clin Endocrinol Metab. 2010 May;95(5):2391-8.
86. Indra T. Left ventricle remodeling in men with moderate to severe volume-dependent hypertension. J Renin Angiotensin Aldosterone Syst. 2012 Dec;13(4):426-34.
87. Kozàkovà M. Myocardial ultrasonic backscatter in hypertension: relation to aldosterone and endothelin. Hypertension. 2003 Feb;41(2):230-6.
88. Matrozova J. Fasting plasma glucose and serum lipids in patients with primary aldosteronism: a controlled cross-sectional study. Hypertension. 2009 Apr;53(4):605-10.
89. Matsumura K. Role of aldosterone in left ventricular hypertrophy in hypertension. Am J Hypertens. 2006 Jan;19(1):13-8.
90. Maule S. QT interval in patients with primary aldosteronism and low-renin essential hypertension. J Hypertens. 2006 Dec;24(12):2459-64.
91. Milliez P, Evidence for an increased rate of cardiovascular events in patients with primary aldosteronism. J Am Coll Cardiol. 2005 Apr 19;45(8):1243-8.
92. Monticone S. Prevalence and Clinical Manifestations of Primary Aldosteronism Encountered in Primary Care Practice. J Am Coll Cardiol. 2017 Apr 11;69(14):1811-1820.
93. Muiesan ML. Structural changes in small resistance arteries and left ventricular geometry in patients with primary and secondary hypertension. J Hypertens. 2002 Jul;20(7):1439-44.
94. Muiesan ML. Inappropriate left ventricular mass in patients with primary aldosteronism. Hypertension. 2008 Sep;52(3):529-34.
95. Murata M. Plasma aldosterone level within the normal range is less associated with cardiovascular and cerebrovascular risk in primary aldosteronism. J Hypertens. 2017 May;35(5):1079-1085.
96. Pimenta E. Cardiac dimensions are largely determined by dietary salt in patients with primary aldosteronism: results of a case-control study. J Clin Endocrinol Metab. 2011 Sep;96(9):2813-20.
97. Rizzoni D. Relations between cardiac and vascular structure in patients with primary and secondary hypertension. J Am Coll Cardiol. 1998 Oct;32(4):985-92.
98. Savard S. Cardiovascular complications associated with primary aldosteronism: a controlled cross-sectional study. Hypertension. 2013 Aug;62(2):331-6.
99. Somlóová Z. The prevalence of metabolic syndrome and its components in two main types of primary aldosteronism. J Hum Hypertens. 2010 Oct;24(10):625-30.
100. Takeda R. Vascular complications in patients with aldosterone producing adenoma in Japan: comparative study with essential hypertension. The Research Committee of Disorders of Adrenal Hormones in Japan. J Endocrinol Invest. 1995 May;18(5):370-3.
101. Tanabe A. Left ventricular hypertrophy is more prominent in patients with primary aldosteronism than in patients with other types of secondary hypertension. Hypertens Res. 1997 Jun;20(2):85-90.
102. Widimský J Jr. Can primary hyperaldosteronism be considered as a specific form of diabetes mellitus? [Physiol Res.](https://www.ncbi.nlm.nih.gov/pubmed/11829322) 2001;50(6):603-7.
103. Mulatero P. Long-term cardio- and cerebrovascular events in patients with primary aldosteronism. J Clin Endocrinol Metab. 2013 Dec;98(12):4826-33.
104. Wu VC. Risk of new-onset diabetes mellitus in primary aldosteronism: a population study over 5 years. J Hypertens. 2017 Aug;35(8):1698-1708.
